# Sirtuin 1 and Sirtuin 3 in Granulosa Cell Tumors

**DOI:** 10.3390/ijms22042047

**Published:** 2021-02-19

**Authors:** Nina Schmid, Kim-Gwendolyn Dietrich, Ignasi Forne, Alexander Burges, Magdalena Szymanska, Rina Meidan, Doris Mayr, Artur Mayerhofer

**Affiliations:** 1 Cell Biology—Anatomy III, Biomedical Center (BMC), LMU München, Großhaderner Straße 9, 82152 Martinsried, Germany; nina.schmid@bmc.med.lmu.de (N.S.); kim.dietrich@bmc.med.lmu.de (K.-G.D.); 2 Protein Analysis Unit, Biomedical Center (BMC), LMU München, Großhaderner Straße 9, 82152 Martinsried, Germany; ignasi.forne@lrz.uni-muenchen.de; 3 Department of Obstetrics and Gynecology, University Hospital, LMU Munich, 81377 Munich, Germany; alexander.burges@med.uni-muenchen.de; 4 Institute of Animal Reproduction and Food Research of the Polish Academy of Sciences, Tuwima 10, 10-748 Olsztyn, Poland; m.szymanska@pan.olsztyn.pl; 5 Department of Animal Sciences, The Robert H. Smith Faculty of Agriculture, Food and Environment, The Hebrew University of Jerusalem, Rehovot 761001, Israel; rina.meidan@mail.huji.ac.il; 6 Institute of Pathology, University Hospital, LMU Munich, Thalkirchner Str. 36, 80377 Munich, Germany; doris.mayr@med.uni-muenchen.de

**Keywords:** granulosa cell tumor, KGN, sirtuin 1, sirtuin 3, siRNA silencing, EX 527

## Abstract

Sirtuins (SIRTs) are NAD^+^-dependent deacetylases that regulate proliferation and cell death. In the human ovary, granulosa cells express sirtuin 1 (SIRT1), which has also been detected in human tumors derived from granulosa cells, i.e., granulosa cell tumors (GCTs), and in KGN cells. KGN cells are an established cellular model for the majority of GCTs and were used to explore the role of SIRT1. The SIRT1 activator SRT2104 increased cell proliferation. By contrast, the inhibitor EX527 reduced cell numbers, without inducing apoptosis. These results were supported by the outcome of siRNA-mediated silencing studies. A tissue microarray containing 92 GCTs revealed nuclear and/or cytoplasmic SIRT1 staining in the majority of the samples, and also, SIRT2-7 were detected in most samples. The expression of SIRT1–7 was not correlated with the survival of the patients; however, SIRT3 and SIRT7 expression was significantly correlated with the proliferation marker Ki-67, implying roles in tumor cell proliferation. SIRT3 was identified by a proteomic analysis as the most abundant SIRT in KGN. The results of the siRNA-silencing experiments indicate involvement of SIRT3 in proliferation. Thus, several SIRTs are expressed by GCTs, and SIRT1 and SIRT3 are involved in the growth regulation of KGN. If transferable to GCTs, these SIRTs may represent novel drug targets.

## 1. Introduction

Mammalian sirtuins (silent information regulator; SIRTs) are a group of seven nicotinamide adenine dinucleotide (NAD)-dependent deacetylases that are located in human cells, typically in the nucleus (SIRT1, SIRT6 and SIRT7), cytosol (SIRT2) and mitochondria (SIRT3, SIRT4 and SIRT5) [1]. SIRTs are involved in a plethora of cellular processes, e.g., metabolism, inflammation, stress responses, aging, proliferation and cancer [2]. 

The female gonad is a dynamic organ. For example, ovarian follicles can grow, differentiate or undergo atresia. SIRT1, but also SIRT3 and SIRT5, are expressed by follicular granulosa cells (GCs), the nurse cells of the oocyte, in different mammals (e.g., humans, cattle, pigs and rats) at various stages of follicular development [3,4,5,6,7]. Evidence has accumulated specifically pinpointing SIRT1 in the control of GC proliferation, apoptosis and secretory activity [3,4,8,9]. Recently, a role in luteinized human GCs was addressed, and for example, evidence for a cAMP-driven expression of SIRT1 was provided [10,11]. Additionally, the SIRT-expression profiles in these cells differ between normal donors and infertile women [12], suggesting involvement in functions related to fertility and infertility.

Follicular GCs are also the origin of human GC tumors (GCTs), which can develop prepubertally (juvenile GCT) or after menopause (adult GCT) [13]. GCTs are distinct from epithelial ovarian cancers and comprise a group of about 5% of all ovarian tumors [14]. They grow indolently and have a high recurrence rate [13]. Despite advances leading to a better understanding of GCT biology, the treatment strategies for GCT patients are still dominated by surgery and re-surgery; systemic chemotherapy is of limited success due to the low tumor proliferation [15,16,17]. Indeed, a high percentage of all the patients, who suffer from aggressive, recurrent GCTs eventually die from their disease [13]. Therefore, new and targeted treatment strategies are needed.

There is growing evidence for a role of SIRTs and SIRT1, in particular, in tumorigenesis [2,18]. For example, SIRT1 activation was shown to inhibit the growth of several cancers (colon, prostate and lymphoma) [19]. Such a role appears to depend, however, on the cell type and context. Furthermore, another member of the family, SIRT3, has a dual role in cancer, as it can act as a tumor suppressor and promotor. SIRT3 is mainly localized in the mitochondrial matrix and plays a key role in the regulation of mitochondrial metabolism and the maintenance of mitochondrial function [20]. Roles of SIRTs in ovarian cancer, namely, epithelial cancer, have been suggested [21,22]. The existing data on the expression and prognostic value of SIRTs were recently summarized by Sun et al. [23]. This comprehensive bioinformatic analysis revealed that SIRT1–4, 6 and 7 might serve as new prognostic biomarkers for epithelial ovarian cancer. Furthermore, the study also concluded that SIRT5 may be a potential target for precision therapy for patients. The study cautioned that additional studies are required to improve the understanding of the roles of SIRTs in epithelial ovarian cancers and to test the mentioned assumptions.

Information on the expression of SIRTs and their possible roles in GCTs remains circumstantial. KGN and COV434, i.e., cell lines derived from GCTs, used in some related studies [4,7,24], express SIRT1. In particular, KGN cells [25] are widely used as an in vitro cell culture model of common, adult GCTs [26,27]. As in our previous studies [15,28], we used KGN cells in the present study. Furthermore, samples from patients and a tissue microarray (TMA) were available to explore whether and how SIRTs may be involved in the growth of GCTs. Here, we provide evidence that all SIRTs are expressed by GCTs. For our cellular study in KGN, we focused on SIRT1 and SIRT3. The results obtained indicate that SIRT1 and SIRT3 are directly involved in cell proliferation, implying that they may represent promising possible targets for personalized therapies for GCT patients.

## 2. Results

### 2.1. SIRT1 in Granulosa Cell Tumors and KGN

**Figure 1:**
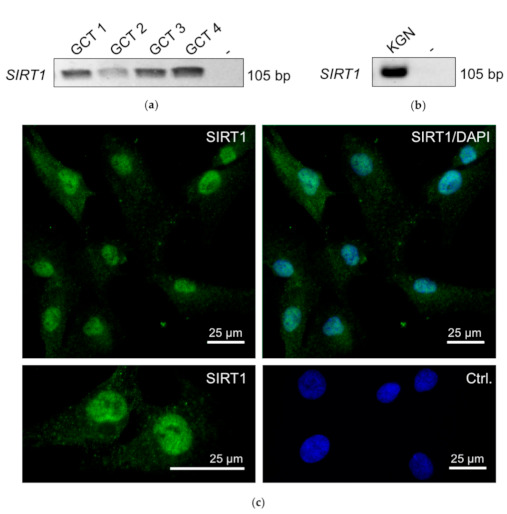
RT-PCR revealed *SIRT1* mRNA (single band of 105 bp) in granulosa cell tumor (GCT) samples from four patients (**a**). Non template (-) control was negative (instead of cDNA, H_2_O was used). (**b**) *SIRT1* was also detected in KGNs (RT-PCR). (**c**) Micrograph of immunofluorescence staining of SIRT1 in KGN. Upper left: SIRT1 staining (green) was found in the nuclei; upper right micrograph: SIRT1 staining merged with DAPI (blue). Left lower panel: higher magnification of the SIRT1 staining. Left lower panel: control: omission of the first antibody merged with DAPI. Bars represent 25 µm.

An RT-PCR screening of GCTs from four patients showed expression of *SIRT1* (Figure 1a), which was also readily detected in the GCT cell line KGN (Figure 1b). Immunofluorescence staining detected SIRT1 within the nucleus and partly in the cytoplasm of KGN cells (Figure 1c).

### 2.2. SIRT1 Activator SRT2104 Affects KGN Cells

**Figure 2:**
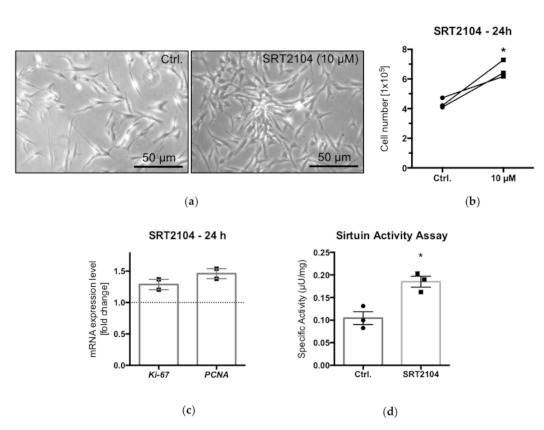
Live cell images of cultured KGN, under control conditions (Ctrl) and upon treatment with the SIRT1 activator SRT2104 (10 µM) for 24 h: higher cell density in the SRT2104 group (**a**). Bars indicate 50 µm. The measurement of cell numbers after SIRT1 activator treatment showed significantly increased cell numbers (**b**) (*n* = 3; * *p* < 0.05, paired *t*-test). Results of qPCR measurements of known proliferation markers, Ki-67 and PCNA; results are normalized to control conditions (**c**). Bars indicate means and SEMs (*n* = 2). Results of a SIRT activity assay revealed significantly increased deacetylation activity upon SIRT1 activator (10 µM) treatment (**d**) (*n* = 3; * *p* < 0.05, unpaired *t*-test).

A specific activator of SIRT1, SRT2104, was used to explore the consequences of activation on KGN viability. We observed significantly increased cell counts (Figure 2a,b) compared to untreated controls after 24 h. Increased mRNA levels for the proliferation markers *Ki-67* and *PCNA* indicated that the activation of proliferation is the underlying mode of action of SRT2104 (Figure 2c). To further examine the underlying mechanism, a SIRT activity assay was performed. The results show that deacetylation activity significantly increased upon SIRT1 activator treatment (Figure 2d). 

### 2.3. SIRT1 Blocker EX 527 Affects KGN Cells

**Figure 3:**
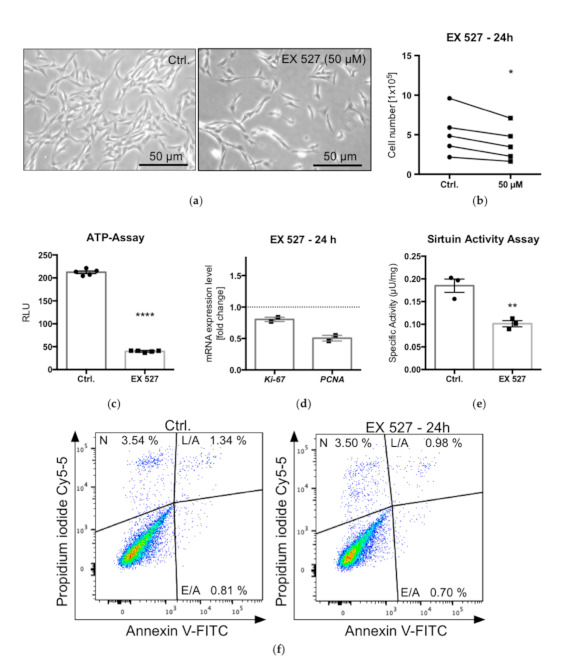
Live cell images of cultured KGN under control conditions (Ctrl) and upon treatment with EX 527 (50 µM) for 24 h: visibly lower cell number after SIRT1 blockage (**a**). Results of cell counting: the treatment with EX 527 resulted in significantly decreased cell numbers (*n* = 5; * *p* < 0.05; paired *t*-test) (**b**). ATP assay after EX 527 treatment (50 µM, 24 h) showed significantly reduced ATP levels in treated cells. Bars represent means and SEMs (**c**) (*n* = 5; * *p* < 0.5, ** *p* < 0.05, **** *p* < 0.0005, unpaired *t*-test). Results of qPCR analysis of proliferation markers *Ki-67* and *PCNA,* normalized to control conditions (**d**) (means and SEMs; *n* = 2). Results of SIRT activity assay indicated significantly reduced deacetylation activity upon blocker (EX 527 at 50 µM) treatment (**e**) (*n* = 3; unpaired t-test). Effects of EX 527 (50 µM, 24 h) on apoptosis and necrosis: FACS analysis of KGN (*n* = 2), double stained with Annexin V and propidium iodide (PI) (**f**). E/A indicates early apoptotic cells (single stained with Annexin V), L/A indicates late apoptotic cells (double stained with Annexin V and PI) and N indicates necrotic cells (single stained with PI).

In contrast to the activator, the SIRT1 blocker EX 527 reduced cell numbers (Figure 3a,b) and ATP levels, which are hallmarks of metabolically active cells (Figure 3c); both were significantly lower compared to untreated controls after 24 h. Furthermore, the qPCR analysis of *Ki-67* and *PCNA* showed reduced mRNA levels (Figure 3d), indicating that EX 527 reduced KGN cell proliferation. The results of a SIRT activity assay showed significantly reduced deacetylation activity upon EX 527 treatment (Figure 3e). To determine whether the action of EX 527 is due to increased cell death events, a FACS analysis was performed. Cells were stained with Annexin V, an established apoptosis marker, and propidium iodide, which stains necrotic cells. There was no evidence for the induction of apoptosis or necrosis as a consequence of the treatment with the SIRT1 blocker (Figure 3f). 

### 2.4. Consequences of Downregulation of SIRT1

**Figure 4:**
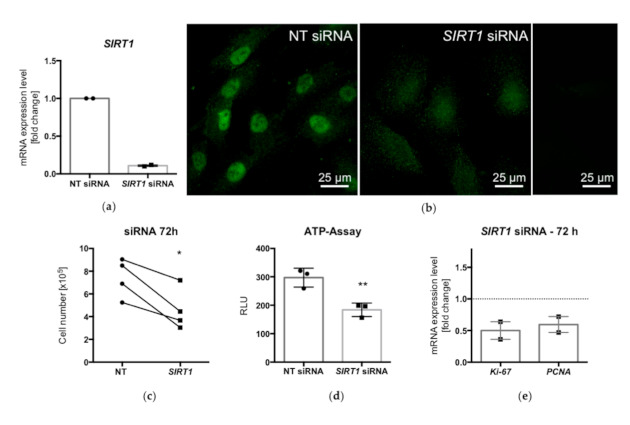
qPCR analysis showed decreased mRNA levels of *SIRT1* after siRNA treatment for 72 h (**a**). Immunofluorescence of KGN after siRNA treatment (72 h): while in nontarget (NT) siRNA-treated cells, prominent SIRT1 staining (green) was observed mainly in the nucleus, silencing of SIRT1 strongly reduced cellular staining (**b**). Ctrl., control experiment (omission of first antibody). Cell numbers (*n* = 4) and ATP levels (*n* = 3) were significantly reduced after siRNA silencing (**c**,**d**) (* *p* < 0.05, ** *p* < 0.005, *t*-test). The levels of the proliferation markers *Ki-67* and *PCNA* were decreased in SIRT1-siRNA-treated KGN, as shown by qPCR analysis (**e**) (means and SEMs; *n* = 2).

To further test the role of SIRT1 in cell growth, a siRNA-silencing experiment was carried out, aiming at the downregulation of endogenous SIRT1 levels in KGN. A qPCR analysis showed a reduction of *SIRT1* levels (Figure 4a). Immunofluorescence staining (72 h post-transfection) indicated that the predominately nuclear presence of SIRT1, which was still seen in the NT siRNA control, was strongly reduced (Figure 4b). The *SIRT1* downregulation resulted in lower cell numbers (found in four out of five experiments; Figure 4c) and lower ATP levels (three additional experiments; Figure 4d). Furthermore, qPCR analysis indicated that *Ki-67* and *PCNA* levels were decreased (Figure 4e). The results are consistent with the reduced proliferation rate associated with lower SIRT1 levels.

### 2.5. Sirtuin Expression in Granulosa Cell Tumors

**Figure 5:**
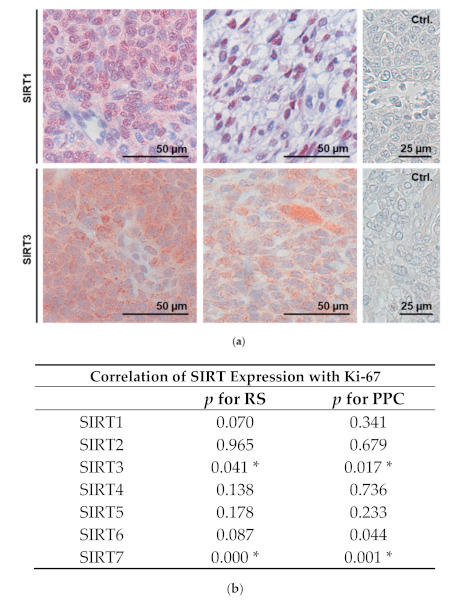
Immunohistochemical staining of SIRT1 and SIRT3 in GCTs (**a**). SIRT1 could be detected in the cytoplasm (left) and/or the nucleus (right), while SIRT3 staining pattern was punctured and found only in the cytoplasm (Ctrl., omission of first antibody). (**b**) Correlation of SIRT expression with proliferation (Ki-67) was calculated for both Remmele scores (RSs) and percentages of positive cells (PPCs). Results of the chi-square test are given, and * *p* < 0.05 was assumed to be significant. Note that for SIRT3 and SIRT7, a higher expression correlated with a higher Ki-67 expression (for evaluation of RS and PPC for calculating the correlation of SIRT1–7 expression with proliferation (Ki-67), see Supplementary Table S2).

A tissue microarray (TMA) containing 92 GCT samples was used to monitor the expression of SIRT1 by immunohistochemistry. In total, 72 (78%) of the 92 samples could be evaluated. The evaluation was carried out by an experienced gynecopathologist (D.M.). Of the 72 samples, 24 were not stained; the others showed either nuclear and/or cytoplasmic staining of SIRT1 (Figure 5a). When the patients’ histories and survival data were taken into account, the staining for SIRT1 (the presence or absence of immunoreactivity) in the respective GCT samples did not statistically significantly correlate with survival (data not shown). 

The TMA was also used to examine proliferation events in the GCT samples using Ki-67 immunohistochemistry. Out of the 92 samples, 91 could be evaluated. Most cases (51%) showed a moderate proliferation rate of 5–10% (Supplementary Table S1). For the SIRT1-positive samples, the trend of higher Ki-67 expression (i.e., the proliferation rate) did not reach statistical significance (*p* = 0.07), even when the Remmele scoring was taken into consideration (Supplementary Table S2). 

Next, the TMA was used to screen for the expression of the other mammalian SIRT family members (SIRT2–7). The analyzability of the immunohistochemical staining is shown in Supplementary Table S3. The immunohistochemistry revealed that all the SIRTs could be detected in most of the tissue samples (71−77%), but as for SIRT1, its expression did not significantly correlate with the survival of the patients. However, the expression of SIRT3 and SIRT7 was significantly correlated with Ki-67 levels (Figure 5b). 

SIRT3 protein was robustly expressed in the analyzed GCT samples; the staining pattern was punctured and only found within the cytoplasm (Figure 5a).

### 2.6. Sirtuin Expression in KGN Cells

**Figure 6:**
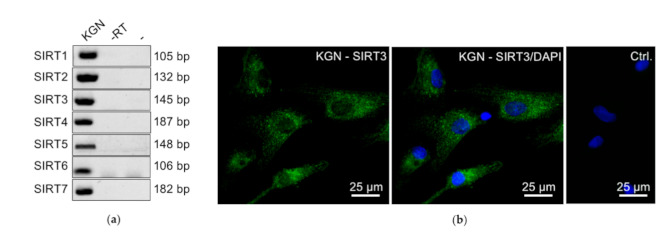
Results of RT-PCR analyses showing expression of *SIRT1–7* in KGN cells (**a**). Immunofluorescence of SIRT3 (green) in KGN showed a prominent punctuate staining pattern in the cytosol (**b**) (Ctrl., control experiments, omission of first antibody; only nuclei are stained with DAPI (blue)).

KGN cells were screened for the expression of all the SIRT family members. RT-PCR analysis revealed the expression of all the SIRTs at the mRNA level (Figure 6a). The proteome analysis of the KGN cells detected SIRT3 (in two out of three samples) among the more than 4900 identified proteins, while the other SIRT proteins were below the detection limit of the LC-MS method (Supplementary Table S4). 

The expression of SIRT3 and SIRT7 in GCTs according to the TMA and their positive correlation with proliferation in GCTs may imply roles in the regulation of cell proliferation. The detection of SIRT3 in KGN by mass spectrometry led us to focus on this SIRT member and its possible roles in KGN proliferation. SIRT3 protein is present in the cytoplasm of KGN cells, as detected by immunofluorescence (Figure 6b), a result in line with the expression in situ, in GCTs (Figure 5a). 

### 2.7. siRNA Silencing of SIRT3 in KGN Cells

**Figure 7:**
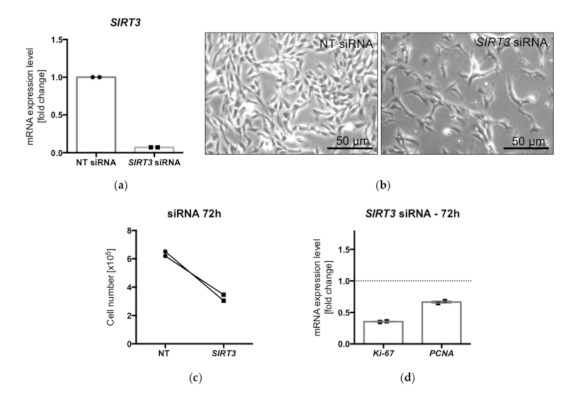
The levels of *SIRT3* were reduced after siRNA silencing of SIRT3 compared to NT siRNA controls (*n* = 2) (**a**). Live cell images of cultured KGN, under control conditions (nontarget = NT siRNA) and after siRNA silencing of SIRT3 (**b**). Lower cell counts after transfection (**c**) and reduced levels of the proliferation markers *Ki-67* and *PCNA* (**d**) (*n* = 2).

To our knowledge, there are no specific pharmacological tools available for specific modulation of SIRT3 activity. Thus, a siRNA-mediated silencing approach was implemented to knock down SIRT3 levels. qPCR revealed the successful downregulation of SIRT3 in KGN 72 h post-transfection (Figure 7a). Reduced SIRT3 levels were correlated with reduced cell proliferation. Visibly fewer cells (Figure 7b) and reduced cell counts (Figure 7c) were detected, compared to nontarget siRNA control conditions. Furthermore, the levels of *Ki-67* and *PCNA* were decreased (Figure 7d). 

## 3. Discussion

To our knowledge, the role of the SIRT family members in human GCTs is poorly understood [7]. The present study identified all SIRTs in human GCT samples, as well as in the cellular model KGN. The evaluation of a TMA linked the expression of SIRT3 and SIRT7 to high proliferation rates for GCTs. While the expression of SIRTs, in general, was not correlated with the survival of the patients, cellular studies performed in KGN support a role of SIRT1 and SIRT3 in the regulation of cell proliferation.

KGN and COV434 are two GCT cell lines that are being widely used as cell culture models for GCs, in general [7] and also for GCTs, in particular. Both were reported to express SIRT1, yet its roles are not well known. For example, a study by Yi et al. [24] focused on polycystic ovarian syndrome (PCOS), and KGN were used to specifically study the role of SIRT1 in mitophagy. Han et al. [4] used COV434 and performed SIRT1 knockdown studies. They concluded that SIRT1 might play an inhibitory role in apoptosis in GCs, possibly by sensing and regulating the ERK1/2 pathway.

SIRT1 was detected in GCTs (Figure 1a and Figure 5a), and thus, we implemented studies in KGN cells, which are considered to be a model for the majority of all GCTs, i.e., adult GCTs, and manipulated SIRT1 activity, using pharmacological tools and siRNA. The selective activator SRT2104 enhanced proliferation, while EX 527, a potent and selective SIRT1 inhibitor [29], reduced cell numbers. There was no evidence for cell death-promoting actions of EX 527. A SIRT activity assay, which measures deacetylase activity, i.e., the genuine activity of SIRTs, was also performed, and the results indicate that SRT2104 and EX 527 act to increase or decrease, respectively, deacetylation activity in KGN cells. The results document the specificity of both the activator and the blocker used and provide further mechanistic support for the involvement of SIRT1 in the regulation of the cell proliferation of KGN. This result is in general agreement with previous studies [7], e.g., in porcine GCs, yet it contrasts to studies in some other tumor cells [19], implying cell-specific actions. ln the mentioned study in GCs, transfection with *SIRT1* cDNA enhanced the expression of proliferation markers, along with a decrease in NF-κB (a SIRT1 substrate), in response to FSH stimulation [3,4,8]. EX 527 (also known as Selisistat), with an IC50 of 38 nM, in a cell-free assay exhibited >200-fold selectivity against SIRT2 and SIRT3. As side effects cannot be completely ruled out, we also downregulated SIRT1 via siRNA, which resulted in the reduced proliferation of KGN. This result is in line with the data of the pharmacological inhibition. Selective SIRT activators and inhibitors are being identified and investigated for therapeutic use in various diseases [29,30]. It became clear that depending on the cell type and the functional relation, SIRT activators can also serve as drugs in cancer therapy, as SIRTs can act as tumor suppressors and promotors [31]. With regard to GCTs, however, the cellular studies in KGN indicated a stimulatory role of SIRT1 activation in proliferation. The inhibition of SIRT1, in contrast, reduced KGN proliferation. Hence, if applicable to the in vivo situation of GCTs, SIRT1 inhibition might be a novel approach to interfere with GCT growth.

The in vivo situation was monitored using human GCT samples. SIRT1 expression or its absence did not correlate with the survival of the patients. Although a trend became apparent, it also did not correlate with cell proliferation (indicated by Ki-67-positive cells) in the tumor samples. Obviously, the limited sample size (92 GCTs in total) may not allow one to draw final conclusions as to whether the absence of SIRT1 may be a good prognostic marker, as we expected from the cellular studies in KGN. While the results did not support a dominating role of SIRT1 expression in the in vivo regulation of GCT growth per se, other factors must also be considered, among others, the further SIRT family members [32]. 

All the SIRTs (1–7) were indeed detected in GCTs, based on the immunohistochemical analyses with the TMA. However, none of them was correlated with patients’ survival. SIRT3 and SIRT7 expression was correlated with high proliferation rates in the GCT samples, and these two SIRT family members have also been implicated in different cancers [33,34,35]. With regard to SIRT3, it can be stated that in some types of cancer, SIRT3 appears to function as a tumor promoter. However, other studies have described SIRT3 as a tumor suppressor with the ability to trigger cell death under stress conditions [33]. Therefore, SIRT3, like other SIRTs, is assumed to have a dual role in cancer cells. 

All the members of the SIRT family were detected at the mRNA level in KGN. However, their abundances varied. Only SIRT3 was detected in two of the three samples analyzed by mass spectrometry, a result probably due to the lower sensitivity of this method compared to RT-PCR. The mass spectrometry results do not rule out the expression of the other SIRT family members at the protein level, though. Especially, our data (including immunocytochemistry) clearly indicated the presence of SIRT1. Nevertheless, based on the results of the proteomic analysis, SIRT3 likely represents the most abundant SIRT in KGN.

SIRT1 was reported to cooperate with SIRT3 in human GCs [7]; thus, the involvement of SIRT3 in the cell proliferation of KGN was explored. Of note, the pharmacological tools for specifically interfering with SIRT3 are very limited [29] to our knowledge. Hence, a siRNA-mediated downregulation of *SIRT3* was performed. The results, i.e., reduced cell proliferation, implicate SIRT3 in the regulation of KGN cell growth. 

Taken together, the results show that all the SIRT family (SIRT1–7) members are expressed by GCTs. The expression levels of SIRT3 and SIRT7 were correlated with high Ki-67 expression, i.e., a high proliferation rate, in GCT samples, implying roles in tumor cell proliferation. Cellular studies in KGN allowed the conclusion that SIRT1 and SIRT3 are directly involved in the regulation of cell proliferation. 

Additional studies with more GCT patient samples are now required to further put these results to the test. SIRTs are multifunctional proteins, and besides proliferation, aggressiveness and metastatic potential should also be examined. As suggested in other tumors [23], such follow-up studies would be important to answer the question of whether the modulation of SIRT activity could be a new target for developing more personalized therapies for GCTs.

## 4. Materials and Methods

### 4.1. Human Granulosa Cell Tumor (GCT) Samples

RNA was isolated from four patient samples (aged 41, 53, 66 and 31) and used for RT-PCR analysis, as described previously [15]. The Institute of Pathology at the LMU Munich diagnosed the GCTs, and an experienced gynecopathologist (D.M.) confirmed the diagnoses. The study was approved (project 390-15) by the LMU ethical committee. 

### 4.2. Tissue Microarray (TMA) and Immunohistochemistry

TMAs were assembled from archived, no-longer-used residual paraffin material, as described earlier [28]. All the cases were pseudoanonymized. All the patients had undergone surgery at the LMU Women’s Hospital (Department of Gynecology, University of Munich, Germany) and were diagnosed at the Institute for Pathology, LMU, Munich, Germany. An experienced gynecopathologist (D.M.) confirmed the diagnoses. In 45 cases, it was the primary tumor; in 47 cases, it was a recurrence with a primary tumor that had been operated on outside the clinic. The ages of the patients at the time of diagnosis ranged from 16 to 89 years. Thirty-three cases (73%) were classified as pT1; four cases (8.9%), as pT2; and eight cases (17.8%), as pT3. Small tissue biopsies (*n* = 92) were taken from representative regions of larger paraffin-embedded tumor samples and were arrayed into a recipient paraffin block, using an MTA-1 (Manual Tissue Arrayer) from Beecher Instruments, Sun Prairie, WI, USA. 

The staining procedures were conducted as previously described [28]. The antibodies used for the study are listed in Supplementary Table S5. Sections were counterstained with hematoxylin and visualized using a Zeiss Axioplan microscope (Carl Zeiss Microscopy, Jena Germany) with a Jenoptik camera (Progres Gryphax Arktur; Jenoptik, Jena, Germany). The total number of GCT samples in the array was 92; thereof, 87 were of the adult and five of the juvenile type. For the evaluation, two different scoring systems were used: the percentage of positive cells (PPC), with 5 groups possible (0 = 0%, 1 = <10%, 2 = 10−50%, 3 = 51–80%, and 4 = >80%), and the Remmele score (RS). The RS grading included the same groups of positive cells multiplied by the staining intensity (1 = low, 2 = middle, and 3 = high); results of 0–12 points were possible.

### 4.3. Cell Culture and Functional Studies of KGN

KGN is a human ovarian granulosa-like tumor cell line, derived from a GCT patient [25] bearing the common FOXL2 mutation, present in 97% of all GCTs [26,27]. It was obtained from the RIKEN BioResource Center, Japan. The KGN culture conditions were described recently [15,36]. In brief, these cells were cultured in Dulbecco’s Modified Eagle Medium/Nutrient Mixture F-12 (DMEM/F-12, Thermo Fisher Scientific, Waltham, MA, USA) complemented with 10% Fetal Calf Serum (FCS) (Capricorn Scientific GmbH, Ebsdorfergrund, Germany) and 0.1 µg/mL Normocin (InvivoGen Europe, Toulouse, France) and incubated at 37 °C with 5% CO_2_. 

### 4.4. Treatment of Cells

The medium was changed to serum-free and antibiotic-free DMEM/F12 two hours before the experiments. The cells were treated with either 10 µM SRT2140 (Absource Diagnostics GmbH, Munich, Germany) dissolved in dimethyl sulfoxide (DMSO) or 50 µM EX 527 (Bio-Techne GmbH, Wiesbaden-Nordenstadt, Germany) dissolved in EtOH for 24 h. For each experiment, a solvent control incubation was included.

### 4.5. Cell Viability Measurement by ATP Assay and Cell Counting

The viability of the cells was estimated by measuring the cellular ATP level, a sign for metabolically active cells, using the CellTiter-Glo Luminescent Cell Viability Assay (Promega, Madison, WI, USA) according to the manufacturer’s instructions and as described previously [37]. In brief, 10^4^ cells/well were seeded on a 96-well microtiter plate (white) and cultured overnight. KGN were treated with EX 527 (50 µM) for 24 h (*n* = 5). The luminescence signal was proportional to the ATP level within the well and detected by a luminometer (FLUOstar Omega; BMG LABTECH, Ortenberg, Germany) and quantified as RLUs (relative luminescence units). Cell counting was carried out using the Casy^®^ Cell Counter (OLS OMNI Life Science, Bremen, Germany) as described earlier [38].

### 4.6. Sirtuin Activity Assay

The cells were seeded on cell culture dishes (5 × 10^5^) and treated with EX 527 (50 µM) or SRT2104 (10 µM) for 24 h. Then, the cells were harvested and used for the SIRT Activity Assay Kit (Sigma Aldrich, St. Louis, MO, USA). The assay was performed according to the manufacturer’s instructions. Fluorometric measurement was conducted with a luminometer (FLUOstar Omega) and quantified as specific SIRT activity in µU/mg. The experiments were repeated three times.

### 4.7. Fluorescence-Activated Cell Sorting (FACS)

KGN (3 × 10^5^) were seeded on cell culture dishes and cultured overnight. Next, the cells were incubated for 24 h with EX 527 (50 µM) or the respective control. The commercial Annexin V -FITC Apoptosis Detection Kit (eBioscience^TM^, San Diego, CA, USA) was used to determine occurring apoptosis and was implemented according to the manufacturer’s instructions. FACS analysis was performed at the Core Facility Flow Cytometry at the Biomedical Center of the LMU Munich, as described previously [28].

### 4.8. RT-PCR and qPCR

RT-PCR was performed as described earlier [39]. Total RNA was isolated using the RNeasy Plus Micro Kit (Qiagen, Hilden, Germany). The concentration and purity of the isolated RNA were measured using a NanoDrop^TM^ (Thermo Fisher Scientific). Reverse transcription (RT) was performed using Superscript II (Invitrogen, Carlsbad, CA, USA) and random 15-mer primers (Metabion International, Munich, Germany). Intron-spanning oligonucleotide primers (Supplementary Table S6) were used for amplification. qPCR was carried out as described earlier [40] (final cDNA concentration, 2 ng/reaction). The calculation of the results was performed using the 2^−ΔΔCq^ method. The results were normalized to ribosomal protein L19 (L19). Negative controls were carried out by the omission of reverse transcriptase (RT) and/or non template reactions using H_2_O instead of cDNA. The PCR products were verified by sequencing. 

### 4.9. Immunofluorescence

For immunofluorescence staining, KGN were seeded onto coverslips and incubated overnight. The cells were either fixed with 3.7% formaldehyde and immediately stained or transfected with SIRT1 siRNA and fixed 72 h after transfection. The fixed cells were washed, permeabilized, counterstained with DAPI and mounted, as described previously [41]. The antibodies were diluted in 0.1% Triton X-100/PBS + 5% goat normal serum, SIRT1 at 4 µg/mL (Atlas Antibody, HPA006295) and SIRT3 at 1:100 (Santa Cruz, sc-365175, Dallas, TX, USA). As s secondary antibody for both SIRT1 and SIRT3, a goat anti-rabbit Alexa Fluor 488 1:1000 antibody (Thermo Fisher Scientific, Waltham, MA, USA) was used. A control consisted of normal goat serum instead of the first antibody. Examination was performed on a Zeiss Z1 Axio Observer microscope.

### 4.10. siRNA Transfection

For the transfection studies 1.2 × 10^5^ cells were plated in culture medium (10% FCS and 0.1 µg/mL Normocin) in a 6-well plate and cultured overnight. Within 24 h, the cells were washed twice with PBS and set in culture medium with 1% FCS without antibiotics. Lipofectamine RNAiMAX (13778075, ThermoFisher Scientific, Darmstadt, Germany) was used as a transfection reagent. The transfection was performed according to the manufacturer’s instructions using Opti-MEM (31985070, ThermoFisher Scientific) for complex dilution. The cells were transfected with 10 nM siRNA for the SIRT1 (sequence, [10]) and SIRT3 (Santa Cruz) targets. For every transfection, a nontargeting (NT) siRNA was used as a control (Silencer Select Negative Control #1, ThermoFisher Scientific). The transfection reagent complex was removed after 20 h, and the cells were supplied with new culture medium (1% FCS, no antibiotics). The cells were harvested 72 h after transfection. 

### 4.11. LC-MS/MS

For these experiments, KGN samples (*n* = 3) were processed using the Preomics iST-kit as recommended by the manufacturer. For LC-MS purposes, desalted peptides were injected in a nanoElute system (Bruker) and separated in a 25 cm analytical column (75 µm ID, 1.6 µm C18, IonOpticks) with a 100 min gradient from 2 to 37% acetonitrile in 0.1% formic acid. The effluent from the HPLC was directly electrosprayed into a Qexactive HF (Thermo) operated in data-dependent mode to automatically switch between full-scan MS and MS/MS acquisition. Survey full-scan MS spectra (from *m*/*z* 375 to 1600) were acquired with a resolution R = 60,000 at *m*/*z* 400 (AGC target of 3 × 10^6^). The 10 most intense peptide ions with charge states between 2 and 5 were sequentially isolated to a target value of 1 x 10^5^ and fragmented at 27% normalized collision energy. The typical mass spectrometric conditions were spray voltage, 1.5 kV; no sheath and auxiliary gas flow; heated capillary temperature, 250 °C; and ion selection threshold, 33,000 counts.

MaxQuant 1.6.3.4 was used to identify proteins and quantify by Label Free Quantification (LFQ) with the following parameters: Database, Uniprot_P000005640_Hsapiens_190109.fasta; MS tol, 10 ppm; MS/MS tol, 20 ppm Da; Peptide FDR, 0.1; Protein FDR, 0.01 min; peptide Length, 7; Variable modifications, Oxidation (M); Fixed modifications, Carbamidomethyl (C); Peptides for protein quantitation, razor and unique; Min. peptides, 1; Min. ratio count, 2. Identified proteins were considered as differential according to their MaxQuant LFQ values. The list of all the proteins detected is provided in Supplementary Table S4.

### 4.12. Statistics

The statistics were performed using the GraphPad Prism 6.0 software (GraphPad Software, San Diego, CA, USA). The ATP assay and the SIRT activity assay were analyzed by unpaired *t*-tests (two-tailed). The cell number and Western blot results were evaluated by paired *t*-tests (two-tailed). The TMA analysis was performed using the chi-square test. *p* values < 0.05 were considered significant.

## Data Availability

Original data are with the authors.

## Supplementary Materials

Supplementary materials can be found at https://www.mdpi.com/1422-0067/22/4/2047/s1.

## Abbreviations

ATP	Adenosine triphosphate
DMEM	Dulbecco’s Modified Eagle Medium
DMSO	Dimethyl sulfoxide
E/A	Early apoptotic cells
FACS	Fluorescence-activated cell sorting
FCS	Fetal calf serum
GC	Granulosa cell
GCT	Granulosa cell tumor
Ki-67	Marker of proliferation Ki-67
L/A	Late apoptotic cells
LC-MS	Liquid chromatography–mass spectrometry
N	Necrotic cells
NAD	Nicotinamide adenine dinucleotide
NT	Nontarget
PCNA	Proliferating cell nuclear antigen
PCOS	Polycystic ovarian syndrome
PI	Propidium iodide
PPC	Percentage of positive cells
RS	Remmele score
RT	Real time
SEM	Standard error of the mean
SIRT	Sirtuin, silent information regulator
TMA	Tissue microarray
