# Supplementary material for: Sirtuin 1 and Sirtuin 3 in Granulosa Cell Tumors
Source: Int J Mol Sci. 2021 Feb 19;22(4):2047. doi: 10.3390/ijms22042047 (PMC7923107; doi:10.3390/ijms22042047)
Supplement: Supplementary file 1 [file ijms-22-02047-s001.zip › suppl files sirt/Supplementary tables.docx]

**Supplementary table 1**

| **Ki-67 immunohistochemistry** | | |
| --- | --- | --- |
|  | Number of cases | Percent |
| < 5% | 28 | 30,4 |
| 5%-10% | 47 | 51,1 |
| 11%-20% | 15 | 16,3 |
| > 20% | 1 | 1,1 |
| total | 91 |  |

**Supplementary table 1**: Evaluation of Ki-67 immunohistochemistry, 91 out of 92 (98.9%) cases were analysable. Four groups were formed due to the overall low positivity caused by low proliferation. The reaction is given in %.

**Supplementary table 2**

| **Remmele-score** | | | | | | | | | |
| --- | --- | --- | --- | --- | --- | --- | --- | --- | --- |
|  | 0 | 1 | 2 | 3 | 4 | 6 | 8 | 9 | 12 |
| SIRT1 | 24 | 3 | 18 | 17 | 8 | 2 | 0 | 0 | 0 |
| SIRT2 | 8 | 3 | 15 | 10 | 4 | 17 | 7 | 3 | 4 |
| SIRT3 | 9 | 6 | 22 | 2 | 12 | 10 | 2 | 2 | 2 |
| SIRT4 | 0 | 0 | 7 | 8 | 6 | 21 | 20 | 4 | 3 |
| SIRT5 | 5 | 3 | 8 | 4 | 11 | 18 | 8 | 3 | 5 |
| SIRT6 | 2 | 12 | 28 | 20 | 5 | 0 | 0 | 0 | 0 |
| SIRT7 | 28 | 13 | 22 | 2 | 3 | 1 | 0 | 0 | 0 |

| **Percentage of positive cells** | | | | | |
| --- | --- | --- | --- | --- | --- |
|  | 0 | 1 | 2 | 3 | 4 |
| SIRT1 | 24 | 3 | 16 | 24 | 5 |
| SIRT2 | 8 | 3 | 20 | 29 | 11 |
| SIRT3 | 9 | 6 | 36 | 12 | 4 |
| SIRT4 | 0 | 0 | 10 | 33 | 26 |
| SIRT5 | 5 | 3 | 17 | 25 | 15 |
| SIRT6 | 2 | 12 | 28 | 20 | 5 |
| SIRT7 | 28 | 13 | 25 | 3 | 0 |

**Supplementary table 2:** For evaluation of SIRT1-7, two different systems were used. First, the evaluation only considered the percentage of positive tumor cells (PPC), which were divided into five groups (0%, < 10%, 10-50%, 51-80% and > 80%). Second, the Remmele-score, with which additionally the staining intensity (low, middle, strong) is also taken into account.

**Supplementary table 3**

|  | **Analysable** |
| --- | --- |
| SIRT1 | 72 (78,3%) |
| SIRT2 | 71 (77,2%) |
| SIRT3 | 67 (72,8%) |
| SIRT4 | 69 (75%) |
| SIRT5 | 65 (70,7%) |
| SIRT6 | 67 (72,8%) |
| SIRT7 | 69 (75%) |

**Supplementary table 3:** Analysability of immunohistochemical staining and verification of SIRTs in GCTs: For SIRT1 72 (78,3%) were analyzable, for SIRT2 71 (77,2%), for SIRT3 67 (72,8%), for SIRT4 69 (75%), for SIRT5 65 (70,7%), for SIRT6 67 (72,8%) and for SIRT7 69 (75%).

**Supplementary table 5**

|  | **Manufacturer** | **Dilution** | **Detection system** | **Application** |
| --- | --- | --- | --- | --- |
| Ki-67 | DAKO | 1:150 | Ventrana ultraView Universal DAB Detection Kit | DAB+ |
| SIRT1 | Atlas Antibodies | 1:180 | ImmPRESS Anti-Rabbit IgG Polymer Kit | AEC+ |
| SIRT2 | Atlas Antibodies | 1:100 | ImmPRESS Anti-Rabbit IgG Polymer Kit | DAB+ |
| SIRT3 | Atlas Antibodies | 1:50 | ImmPRESS Anti-Rabbit IgG Polymer Kit | DAB+ |
| SIRT4 | Abcam | 1:120 | MACH 3 Rabbit AP Polymer Detection | Permanent AP Red |
| SIRT5 | Origene | 1:100 | ImmPRESS Anti-Rabbit IgG Polymer Kit | AEC+ |
| SIRT6 | ThermoFisher Scientific | 1:280 | Vectastain ABC-Kit Elite Universal | AEC+ |
| SIRT7 | Atlas Antibodies | 1:350 | ImmPRESS Anti-Rabbit IgG Polymer Kit | AEC+ |

**Supplementary table 5:** List of Antibodies used for the TMA immunohistochemical staining.

**Supplementary table 6**

| **Gene** | **Gene name** | **Reference ID** | **Nucleotide sequence** | **Amplicon size** |
| --- | --- | --- | --- | --- |
| *Ki-67* | Marker of proliferation Ki-67 | NM_002417.5 | 5´-GAGAATCTGTGAATCTGGGTAA-3´  5´-CAGGCTTGCTGAGGGAAT-3´ | 279 |
| *PCNA* | Proliferating cell nuclear protein | NM_182649.2 | 5´-CAAGTAATGTCGATAAAGAGGAGG-3´  5´-GTGTCACCGTTGAAGAGAGTGG-3´ | 126 |
| *RPL19* | Ribosomal protein L19 | NM_000981 | 5′-AGGCACATGGGCATAGGTAA-3′ 5′-CCATGAGAATCCGCTTGTTT-3′ | 199 |
| *SIRT1* | Sirtuin 1 | NM_012238.4 | 5´-CAGTGGCTGGAACAGTGAGA-3´ 5´-AACGATTTGGTGGCAAAAAC-3´ | 105 |
| *SIRT2* | Sirtuin 2 | NM_012237.4 | 5´-GGATGATTATGGGCCTCGGA-3´ 5´-CCAGCTCCTTCTTCCATCCA-3´ | 132 |
| *SIRT3* | Sirtuin 3 | NR_163398.1 | 5´-GAAGCTCATGGAACCTTTGC-3′ 5´-AGAACACAATGTCGGGCTTC-3´ | 145 |
| *SIRT4* | Sirtuin 4 | XM_024448907.1 | 5´-GCTTTGCGTTGACTTTCAGGT-3´ 5´- GAGATTCCTGCCCCAGTCAT-3´ | 187 |
| *SIRT5* | Sirtuin 5 | NM_001376803.1 | 5′-GTCCACACGAAACCAGATTTCCC -3′ 5′-TCCTCTGAAGGTCGGAACACCA -3′ | 148 |
| *SIRT6* | Sirtuin 6 | NM_001321062.1 | 5′-TTGTGGAAGAATGTGCCAAGTG-3′ 5′-CCTTAGCCACGGTGCAGA G-3′ | 106 |
| *SIRT7* | Sirtuin 7 | NM_016538.3 | 5′- TGAAGTCTGTACCTCCTGCG-3′ 5´-CTTCCCAGTTCAAAGGCTGC-3´ | 182 |

**Supplementary table 6:** List of oligonucleotide primer for PCR studies
